# Arts and health as a practice of liminality: Managing the spaces of transformation for social and emotional wellbeing with primary school children

**DOI:** 10.1016/j.healthplace.2012.06.017

**Published:** 2012-11

**Authors:** Sarah Atkinson, Mary Robson

**Affiliations:** Centre for Medical Humanities, Department of Geography, Durham University, Durham, United Kingdom

**Keywords:** Creative arts, Wellbeing, Liminality, Primary schools, Practice

## Abstract

Intervention to enhance wellbeing through participation in the creative arts has a transformative potential, but the spatialities to this are poorly theorised. The paper examines arts-based interventions in two primary schools in which small groups of children are taken out of their everyday classrooms to participate in weekly sessions. The paper argues that such intervention is usefully seen as a practice of liminality, a distinct time and space that needs careful management to realise a transformative potential. Such management involves negotiating multiple sources of tension to balance different modes of power, forms of art practices and permeability of the liminal time-space.

## Highlights

The paper explores how arts-based projects may impact personal social and emotional wellbeing. ► These processes are studied empirically with targeted groups of children in two primary schools. ► Targeted arts-and-health interventions are usefully understood as a practice of liminality. ► Liminality needs careful management to be effective in realising its transformative potential. ► The conceptual distinction between liminal and liminoid spaces retains continued relevance

## 1 Introduction

The argument that engagement in, and with, the creative arts to benefit health and wellbeing is now supported by a growing body of evidence (Staricoff, 2004) and recognised in health policy communities (Arts Council, 2007; Arts Council/Department of Health, 2007; Department of Health, 2006; Fiske, 1999; Karkou and Glasman, 2004). However, exploration of how such impacts may be brought about has been more limited. To date, theorisation of the therapeutic processes of participation in the creative arts is largely grounded in the traditions of psychoanalysis or developmental psychology (Karkou and Sanderson, 2006). These intellectual roots largely neglect the spatial domain and the spaces of transformation thus tend to be invisible (Daykin, 2007; Sagan, 2008). The paper addresses this invisibility by proposing that a renewed engagement with Turner's concept of liminality provides a useful framework for understanding the spatial aspects of the processes and practices through which arts-based interventions in schools can enhance participants’ social and emotional wellbeing. In doing so, the paper also draws attention to the management challenges inherent to arts-based intervention as a practice of liminality. The paper explores these spatial aspects through case studies of arts-based interventions in two English primary schools. These interventions were designed to improve social and emotional wellbeing and were both considered to have been successful in this by the schools involved.

Addressing personal wellbeing in English schools was prioritised and promoted through the national programme for Social and Emotional Aspects of Learning (SEAL, 2007–2011). The SEAL programme drew on Goleman's notion of emotional intelligence (1995) to define its goals through five dimensions: self-awareness, self-regulation in managing feelings, motivation, empathy and social skills. The programme advocated diverse forms of intervention, including through the creative arts, and at various scales, including with targeted groups of children (Humphrey et al., 2008; Roffey, 2008). Selection criteria for targeted participation in schools vary but have included educational needs, social and emotional difficulties or fostering a mix of ‘competencies’. Schools using interventions based in the arts usually contract an artist to run regular sessions with each group over a set period of time. A targeted intervention thus engages children into a time, space and set of activities distinct from the everyday life of the classroom: children are literally in a different space; there is a different mix of children; they are not with the classroom teacher; and they are engaged in activities outside the standard curriculum. The arts practitioner is also external to the school and often to the teaching profession. This removal from the everyday routines in order to effect personal transformation shares much with Turner's elaboration of the liminal and the liminoid (Turner, 1967, 1969, 1974).

### 1.1 Arts, transformation and wellbeing

There are two related professional fields that engage with the creative arts for health which, despite much overlap, can be distinguished by their core practice and theoretical underpinnings. Art therapy treats clients with identified pathologies and is explicitly theorised through a range of psychotherapeutic and developmental traditions (including Jung, 1990; Klein, 1975; Piaget, 1972; Winnicott, 1971). The arts constitute a tool to explore pre-verbal functioning, both to gauge psychological wellbeing and to interact with the inner world through the playful and spontaneous possibilities for self-expression that the arts can enable (Karkou and Sanderson, 2006; Malchiodi, 2011). By contrast, arts-and-health practitioners treat the process of art-making itself as a therapeutic experience that can enhance positive wellbeing. Thus, school interventions, such as the case studies of this paper, are delivered by creative ‘arts-and-health’ practitioners who are primarily practising artists.

The dynamics of how an arts-and-health intervention may afford a therapeutic experience is less well theorised than the practices of the art therapist. Existing research focuses on the social processes through which personal gains are enabled rather than the hidden psychological processes. A dominant theory emerges, albeit implicitly, that participation in the creative arts may build both an inward-looking self-esteem and self-awareness and an outward looking social confidence and connectedness (Clift and Hancox, 2010; Hampshire and Matthijsse, 2010; Hillman, 2002; Parkinson, 2009) which, in turn, open up new narratives through which to construct resilience and make choices (Elliott, 2011; Nakamura and Csikszentmihalyi, 2002; Peerbhoy and Kilroy, 2008). As such, transformation involves the re-imaginings of oneself, one's capacities and one's interrelationships with others, a process of ‘changing our stories’ (Wynne, 1987: 482) which draws on conceptualisations of identity as, at least in part, autobiographical and narrative (Bauer et al., 2006; Singer, 2004; Zahavi, 2007).

However, the emphasis in this approach on openness to alternative narratives of identity may conflict with the dominant model of child development that underpins the national curriculum and initiatives such as SEAL. In this, the child is framed less as a present being than as in a process of becoming, and, moreover, as a becoming that is pre-scripted as progressing through linear and universal developmental stages (Karkou and Sanderson, 2006; Piaget, 1972; Valentine, 2004). Within this framing, intervention through the creative arts aims to support children's personal development in terms of emotional and social management which, in turn, are understood to influence immediate pedagogic goals and longer-term health and wellbeing (NICE, 2008). This framing has been widely critiqued empirically and ideologically. Empirically, children have demonstrated far greater competencies than assumed with their apparent stages of development shaped by their social-cultural context (Valentine, 2004). Ideologically, critics have argued that children should not be understood as only and always becoming but, first and foremost, as being (Aitken, 2001; Holloway and Valentine, 2000). The psychoanalyst Winnicott (1971) also contested a linear standardisation of childhood by emphasising the spatialities of early attachments and separations. More specifically, and resonant with the practices in arts-and-health, Winnicott proposed the concept of transitional space as an arena in which one can safely explore one's agency and play with different ways of being in the world (Kullman, 2010).

Winnicott's emphasis on the spatialities of development complements the attention in arts-and-health to ‘changing our stories’. Combined, these perspectives frame personal wellbeing as emerging through situated and relational effects that are dependent on the mobilisation of resources within different social and spatial contexts (Kesby, 2007). In the spaces of wellbeing approach (Fleuret and Atkinson, 2007), wellbeing is emergent through four interrelated spaces of resource mobilisation: capabilities (Nussbaum, 2000), social integration (Putnam, 2001; Wilkinson and Marmott, 2003), security (Shaw, 2004) and therapeutic processes (Conradson, 2005; Smyth, 2005). Connecting this to Winnicott, the task of the arts-and-health practitioner thus becomes the creation of transitional spaces within which openness is enabled to explore new possibilities for identity and action, spaces in which new resources can be built and mobilised for personal wellbeing. Our proposition, then, is that the arts-based practices that can successfully generate such spaces of wellbeing are usefully explored and conceptualised through Turner's theory of liminality. We have organised the paper so as to integrate conceptual themes, including description of the main tenets of Turner's theory, with the empirical material from our two case studies.

## 2 Two targeted arts-based interventions

The paper draws on case studies in two primary schools over two academic years (October, 2008 to July, 2010) (Yin, 2009). The two interventions were selected from ‘Inside Me’, a programme of arts-based intervention to enhance personal social and emotional wellbeing in six primary and secondary schools in areas of significant social and economic disadvantage in West Yorkshire, UK, implemented by the arts agency Loca and funded by the local health authority. The ‘Inside Me’ programme received ethical approval through the NHS; the study received ethical approval through University procedures compliant with the UK ESRC requirements. Apart from the programme ‘Inside Me’ and the implementing agency, Loca, all names have been changed.

Evaluation of the overall programme showed strong positive impacts, such that an original two-year programme was extended for a further year (Loca, 2009; White and Robson, 2012). In order to explore the processes and practices involved in successful intervention, we selected two primary schools (ages 5–11 years) where the school rated the project as successful in enhancing participants’ personal wellbeing and where the same arts practitioner had been involved throughout. Both case study schools were urban, with the proportion of children eligible for free school meals and those with a statement of special educational needs much higher than the national average. Brightfields has a school population mostly from white British backgrounds whereas Pennington has over a third from minority ethnic backgrounds, including a small group of asylum seekers and refugees. Brightfields opted for interventions using drawing and modelling, led by the arts practitioner Alice and Pennington opted for an intervention through writing, led by the arts practitioner Michael. The practitioners ran and facilitated a range of different activities; we have included only those for which children were purposively selected by staff to form a group which met regularly over at least a term for a morning or afternoon session once a week. More informal and self-selecting drop-in sessions, one-off sessions or intensive sessions with a guest artist have not been included. The overall numbers involved in these interventions were relatively small: Pennington ran eleven different groups in which group size was never larger than six, often only four; Brightfields ran thirteen different groups in which group size ranged from six to ten. A formalised code-of-practice for arts-and-health practitioners has been in development since the ‘Inside Me’ programme, drawing on the experiences from reflexive practice in this and other arts-based projects. The ethos of the intervention, the orientation of the practitioners to the group work as a shared endeavour and the investment to reflexive practice throughout the programme's duration accorded with guides and standards of practice for group work in social work (see, for example, AASWG, 2006). Agreed standards of practice only provide generalised guidance and where practitioners become ‘more preoccupied with protocols, curricula, and manuals that with their group members’ narratives and group processes’, poor practice still results (Gitterman, 2011: 8). The challenge for any practitioner working with groups is to realise joint ownership through a reflexive practice that is sensitive to the specifics of the particular project.

Since our research sought to understand the dynamics of a successful intervention, rather than evaluating the outcomes themselves, our main focus was the practices of the arts practitioner. However, the arts sessions worked with small numbers of children and we were mindful that the presence of a further adult in the sessions would radically change the dynamics. We therefore enrolled the two arts practitioners as both researchers and informants, enjoined to provide a reflexive and detailed account of each session at the time. Practitioners used an open format form which prompted them to record the activities and intentions of the session and reflection on the session dynamics such as how the children had engaged, their interactions and comments, problems in the session and the practitioners’ own learning. The arts practitioners welcomed keeping records, which for them constituted a form of action-research as their reflections fed back into their on-going practice (Reason and Bradbury, 2008).

These accounts were supplemented by our own occasional observations of sessions, visits to the schools and conversations with school staff and children and through conversations with the arts practitioners both within and outside the school. One of the authors was contracted by the ‘Inside Me’ project to co-ordinate reflexive practice with all arts practitioners in the project. In this capacity, she visited the ‘Inside Me’ interventions regularly throughout the two years, met and discussed the interventions with both school staff and the arts practitioners, developed the template with the practitioners to record their activities and their reflections after each session and held ‘salons’ at which arts practitioners met one another to exchange experiences and ideas. The notes and records of her observations, conversations and salons act to triangulate, through corroboration and elaboration (Hammersley and Atkinson, 1983; Creswell, 1998), the archive of material provided by the practitioners’ own records and reflections regarding the activities and experiences of each session.

The written records and reflections by the arts practitioners are analysed through interpretative analytical approaches to qualitative data (Denzin, 1997; Silverman, 2006; Wolcott, 1999). We read through the ‘archive’ several times and made an initial coding of topics, which we grouped into a few broad themes: starting up, including naming and setting rules; collective activities and team building; practices to promote transformation; evidence of shifts; relationships to the wider school—intrusions and sustaining change. This was the stage at which we noticed that our own themes mapped coherently against the elements of liminality.

## 3 Arts-based intervention as liminality

### 3.1 Dislocation and dissolution

The concept of liminality was elaborated by the anthropologists Van Gennep and Turner to describe the central phase of formalised rituals and processes that bring about transformations spiritually or in social status (Van Gennep, 1960; Turner, 1967). Turner, in particular, discussed not only the structural role of rites of passage within societies, but also how the practices and experiences of liminality may provoke transformations in identities and agency. This transformational potential is structured by both emplacement and movement, an indivisibility of time-space:

‘If one attends to the boundary itself, the emphasis becomes spatial, but if one attends to the person making the crossing, the emphasis becomes temporal and processual’ (Grimes, 2006: 113).

Liminality aims to disorient through a ritualised withdrawal from the habits and routines of the everyday social order and the dissolution of existing structures of thought, action and identity.

for me the essence of liminality is to be found in its release from normal constraints, making possible the deconstruction of the “uninteresting” constructions of common sense, the “meaningfulness of ordinary life”. (Turner, 1977: 68).

Liminality thus entails an effective separation from the everyday routines and entry into an alternative social encounter in which different rules, different values and different relations apply.

Each of the targeted groups met for a weekly morning or afternoon session and almost all mixed children from different classes. Given the intermittent nature of participation in new, social groupings, use of a distinct and identifiable space facilitated rapid re-entry each week. Initially, Pennington had no dedicated space and Michael was timetabled into whichever room was available, which he found unsettling. Alice usually had the art-room at Brightfields, but she also noted the unsettling effect when this was not fully available.

‘It was quite a difficult session as the room was being used by another artist … it was a bit distracting’. (Alice, 5/10/09).

Importantly, both schools quickly recognised the need for dedicated spaces:

I think it would be good turn the library into a permanent wellbeing area, so that you can have a space that feels like your own for the project and the school has a special place for this kind of thing. (Headteacher, Brightfields, 07/11/08).

Turnerian liminality consists of a single, protracted period of separation in which transformation occurs as intense turning points. This perception of abrupt transformation has been critiqued as a gendered narrative:

when women recount their own lives, the themes are less climax, conversion, reintegration and triumph, the liminality of reversal or elevation, than continuity. (Walker Bynum, 1991: 32–33).

However, contemporary applications of liminality have modified Turner's characterisation to allow short but repeated periods of separation. Examples include spaces of training (Buckingham et al., 2006), of breastfeeding (Mahon-Daly and Andrews, 2002), of school libraries and learning (Dressman, 1997), of music (Boyce-Tillman, 2009) and of dying and bereavement (Froggatt, 1997). Allowing flexible temporalities to liminality addresses the critique of abrupt transformation by recognising gradual incorporations of new identities. For example, research on the repeated use of on-line spaces to mentor newly qualifying teachers (Cook-Sather, 2006), or to try out identities as new mothers (Madge and O'Connor, 2005) both drew on the concept of liminality to understand the transition and transformation to a new status.

The arts practitioners demarcated the time-space through what were effectively entry rituals: start-up activities in the first session and warm-up routines in subsequent sessions. Many groups were given writing or drawing books during the first session:

‘We have the writing books and no-one else does. It's like a secret.’ (Documented by Michael, 11/11/09).

Sessions started with group activities in a circle, dubbed ‘circle-time’. Alice used a signing in ritual in which the children chose a style of writing and a word to describe how they felt which they all then discussed in the circle-time; Michael did something similar with colours and feelings. The children enjoyed these repetitive routines and Michael himself described this as, ‘A nice ritual too’ (27/4/09). The use of messy and fluid equipment at Brightfields required organised seating but the writing at Pennington allowed flexibility about how and where they sat. The children themselves initiated uses of the space that both differed from classroom practice and effectively created rituals, including a Reading Chair and a listening space:

‘Can we sit on the cushions and listen?’ (Documented by Michael, 26/01/08).

Michael adopted these child-led innovations across subsequent groups; the Reading Chair was particularly popular, relaxed the reader and improved the attention of the listeners. Some children even formalised their own spaces in the room:

‘Jack writes more than has ever done and says, “Can I go and sit in my special writing place?”’ (Michael, 25/11/09).

The small numbers and the explicit attention to confidence-building gave children the freedom to initiate, request and lead activities, a dissolution of the rules and expectations that often adhere to the classroom setting. From the outset, greater familiarity was allowed with the practitioners:

‘I like that we can call you by your name’ (Documented by Alice,19/06/09).

The practitioners themselves intentionally aimed to dissolve established rules of working:

‘I'm going to encourage some word fun in future, some breaking of rules, mild transgression’. (Michael, 21/10/09),

including the inversions of status characteristic of Turnerian liminality:

[she] proceeded to give me orders about what shapes she wanted me to cut out for her. It was great fun, she found it very funny and it was fab to see her so cheeky and relaxed. (Alice, 10/3/10)

Children at Pennington commissioned Michael to write for them on a theme of their choice; one group gave him a hand-writing lesson and, since he joined in with the various tasks, they noted that this was the:

‘first time I've ever seen a teacher doing the thing they've set us’. (Documented by Michael, 12/01/09).

Turner understood the dissolution of rules and inversions of power as disorientating, a process essential to generating openness to the possibilities for transformation and resonant with Winnicott's space of transition (1971). Some children clearly manifest their disorientation, being reluctant to start, wanting to know the rules and anxious to do it right:

‘Shall I put the date? Title?’. (Documented by Michael, 13/01/09). ‘I don't know what to put. I've never written a poem’. (Documented by Michael, 11/11/09).

‘Something I come across again and again is how difficult the children seem to find it to let go and be really wacky with their ideas’. (Alice, 2/10/09).

Alice provoked letting go through quick, timed painting that made impossible any polished product and thus prompted new exploration of self and capacities:

‘it was interesting to see how just the act of timing them focussed their concentration and gave them permission to be free and experimental’. (Alice, 3/11/09).

### 3.2 Communitas and control

Turner's ‘deconstruction of the “uninteresting” constructions of common sense’ (1977: 68) was further enabled by eroding everyday hierarchies in favour of *‘communitas’*, a sociality based on equality and trust (Turner, 1967). Communitas reflects a shift in the relative balance between the different modes of power exercised compared with other spaces. Whilst teachers draw on diverse modes of power to promote successful pedagogic outcomes, the definition and assessment of those outcomes remains with their profession. Moreover, skilled avoidance of overt conflict or disruption is achieved through the normative imposition of habituated forms of conduct, with little tolerance for non-compliance. These modes of power constitute a form of domination, the implementation of which is mediated by those, such as teachers, appointed to structural positions of authority (Allen, 2003). The arts practitioners entered the school in a different structural position, as facilitators rather than instructors and with no predetermined goals other than to facilitate the exploration by participants of their emotions, their interpersonal relationships and their interactions with wider environments. Any problems or deficits attributed to the children in an educational context were largely replaced in the arts sessions by a positive attention to the children's own emotional and imaginative capacities. The children themselves recognised that a space had been created within which they enjoyed a different learning experience:

‘I like words in this group, so why don't I like Literacy in class?’. (Documented by Michael, 16/06/09).

‘It's different from school, it gives you space and you haven't got everyone else rabbiting on’. (Documented by Alice, 19/6/09).

The position and approach of the arts practitioner as facilitator had strong parallels with the contemporary approaches in group and community social work that explicitly aim to establish relationships between social worker and clients based on equality, mutual recognition and joint ownership of the intervention process. The strengths-based perspective takes a pragmatic approach in which intervention identifies, values and builds from existing individual strengths (Early and GlenMaye, 2000; Saleebey, 2006). The arts-based interventions explicitly intended to reveal to children their own capacities and thereby build their confidence and self-esteem. This approach represents an important shift away from problems and deficits, but ignores how power is structured into the institutions and interpersonal relations of society and, as such, does not challenge who defines the pre-set outcome goals of intervention (Gray, 2011). This was evident also in the English school's SEAL programme in which a focus on social and emotional wellbeing was underpinned by societal and pedagogic goals. Feminist and anti-oppressive approaches within social work aim to invert the relationships of oppressive power that structure clients’ lives and their encounters with the institutions of social welfare (Dominelli, 2002, 2006). These approaches centre on practices of facilitation and influence (Zastrow, 2009) in which ‘power with’ is effected through modes of negotiation, seduction and persuasion (Allen, 2003). The arts practitioners similarly manifest an ideal of ‘anti-oppressive’ practice in their aim to work with the children through an egalitarian relationship, the expression of ‘power with’ as opposed to ‘power over’ (Allen, 2003).

Turner's concept of communitas as the emergence of a homogeneous, consensual group has similarly been critiqued as ignoring the dynamics of motivation and power inherent in any group (Eade, 1992; Eade and Sallnow, 1991). Some authors have suggested that contemporary spaces of liminality show so little communitas that they are better conceived as spaces of alternative heterotopia (St John, 2001). However, these applications of liminality tend to be in medium-scale spaces explicitly positioned as alternative, such as festivals or tourist destinations. In the very local and proximate setting of the arts-based small group work, the practitioners recognised the importance of trying to establish a group identity and were mostly successful in generating *‘communitas’* underpinned by bonds of trust through which children,

‘shared things about themselves that I don't think they would ordinarily feel comfortable saying in a large group’. (Alice, 23/09/09).

Michael facilitated group identity through two activities which both defined boundaries and built cohesion: naming the group and collectively setting the session rules. These activities also signalled a mode of ‘power with’ between practitioner and participants expressed through negotiation; the groups demonstrated their sense of both ownership and belonging through affiliation with their group name:

‘Michael, why are you calling us ‘the group’ when we have a name?’. (Documented by Michael, 11/11/09).

The arts activities also built communitas. Both practitioners frequently brainstormed with the group to generate ideas either for a collective product, such as a poem, or to equip children for subsequent work in pairs or individually. This enabled the children to support one another in a new activity but also to explore and express themselves individually:

‘… it is a group effort as they are so inspired by one another yet now more often endeavouring to be original and imaginative’. (Alice, 3/11/09).

However, inverting power relations is difficult; critics have pointed out that in social work, the professional worker retains the power to define what counts as oppression (Wilson and Beresford, 2000). In our interventions, the arts practitioners retained the power to define the limits to inverting power and transgressing everyday codes of conduct. The compelling rhetoric to share ‘power with’ hides a major challenge to the practitioner to resolve tensions related to the exercise of power in managing group dynamics. The arts practitioners in dissolving the everyday codes of behaviour did not aim for an absence of codes but the emergence of different ones, ideally based on anti-oppressive modes of ‘power with’ to facilitate mutual recognition of one another's strengths:

‘in order for good creative work to be done, there has to be respect for the process and for each other’. (Michael, 04/12/08).

But whilst the arts practitioners aimed to facilitate a greater sense of freedom and exploration, they sometimes found that,

‘..they [the children] bounce about in that freedom and I feel walked all over’. (Michael, 13/01/09).

Both Michael and Alice found themselves facing a tension between their own ideal of a shared ‘power with’ the group and the evident need at times to exert authoritative ‘power over’ the group (Allen, 2003). They tried to manage disruptive conflicts through shared group responsibility for negotiating the rules:

‘I always worry about trying to discipline too much but it was good to get a discussion going with the group about what feels reasonable in certain situations’. (Alice, 19/6/09).

‘..must set tone and boundaries straight away. Not my forte and I so bloody hate being Mr. Discipline. …I want to be myself, treat them as grown-ups, but of course they're not and I really have to work hard to achieve it’. (Michael, 13/01/09).

Nonetheless, small groups are easily disrupted and both artists on occasions reluctantly resorted to some authoritarian inputs and sanctions in order to enable non-authoritarian relations to flourish:

‘There are three very anarchic boys in this group and they are proving to make sessions very difficult … Lewis had to be sent back to class early on in the session’. (Alice, 07/11/08).

‘Janey kept mucking about so I told her to go…. I'm glad I did it as otherwise I would have felt completely run over’. (Michael, 13/01/09).

This tension is evident also in guidance on facilitating group social work; Zastrow (2009) both warns that seeking popularity or asserting authority can diminish the capacity of a group, and advises hostile or disruptive behaviour should be confronted firmly. The capacity of the arts-based interventions depended on the arts practitioners’ success in facilitating a balance between different modes of power. Too little authority and the arts sessions would have collapsed into chaos; too much and the liminal time-space would have become indistinguishable from the everyday classroom. In this respect, the arts-and-health practitioners resembled Turner's mentor or guide in the liminal phase more closely than their counterparts in arts therapy for whom maintaining a partnership of ‘power with’ is essential (Karkou and Sanderson, 2006).

### 3.3 Arts practices towards transformation

The arts practitioner further fulfilled Turner's role of mentor and guide by facilitating games, rituals, tasks and transgressive behaviours. Arts activities prompted direct exploration and expression of feelings and identities, indirect explorations of interactions and behaviour in imaginary settings, experimenting with new techniques and producing pieces of art.

Arts activities on occasions provided outlets for anger:

‘They all came in wanting to talk about how angry they were… We listed angry words on the board …… We wrote a very quick angry poem together on the board which I read out in a very angry way. This interested them.’ (Michael, 27/01/09).

‘We took turns throwing the clay down onto the table … and shouted out words. It was brilliant and replaced the angry and frustrated mood with laughter’. (Alice, 7/10/09).

The artists facilitated redirecting anger through a willingness to be spontaneous and exploratory. Brown (2009) emphasises the importance of spontaneity in group work as a capacity that can liberate creativity. Moreover, spontaneity for some children was physical and the physicality of several arts activities suited some children. The small numbers could accommodate children moving around the room, which clearly benefited some of the boys:

‘… but occasionally needs to walk about. He knows he has to do this when frustrated. I know this too now. It's a way of separating himself off for a moment.’ (Michael, 05/05/09).

‘Niall was beginning to be a bit of a handful until we started the movements … It just calmed him down immensely to express himself through movement’ (Alice, 21/1/10).

and, occasionally, the whole group,

‘Very jumpy energy – half-term coming up. In fact what they did was acute and productive but they had to move around’. (Michael, 21/10/09).

Turner's thesis gives little attention to embodiment, despite the physicality of many rituals (St John, 2001). The enabling of alternative, and in the context of the school, transgressive movement and bodily expressions may be key to opening new narratives of identity for these children.

The direct exploration of emotions risked the expression of strong emotions, but in a manner which closed down the possibility to explore these further. This was counterproductive to group coherence and both artists subsequently avoided a direct approach:

‘I really don't want the children to use the session as an excuse to vent all their problems… that happened last year and meant that one or two people were dominating the group in quite a negative mood.’ (Alice, 7/10/09).

Indirect approaches proved more constructive. Michael used other sensory stimuli as metaphors to provoke explorations of emotions including colours, paintings, music and moulding each other's bodies into emotionally expressive shapes. Alice explored identity when decorating coil pots; each child picked three colours to represent themselves when painting the inside and collectively picked one colour to represent each child in the group to paint the outside. Both artists explored relationships with different places. Children created and populated imaginary worlds from which they reflected on feelings and the values of different personal qualities in different situations. Imaginary worlds also allowed the children-creators to explore and invert usual rules, creating a world in which children stayed at home and adults went to school, or in which familiar fictional characters, such as Horrid Henry, worked in unlikely real-world places, such as a zoo. Transgressive practices and inversions of adult-child roles thus enabled new techniques of self-expression and experimentation with new potentials.

‘The kids enjoyed having the freedom to draw huge pictures and after the initial timidness had passed they all created bold and exciting pictures’. (Alice, 3/2/10).

Incorporating the children's own suggestions into the session activities and inverting the expected relations of adult and child, or teacher and pupil, also affirmed the value of their ideas and ways of working:

‘This time I didn't stop them all huddling under a table together doing their writing. I joined them. It became our writing den. Silence, concentration! Fantastic.’ (Michael, 23/02/09).

The project delivered a direct material message to the children of their worth through the provision of high quality arts materials. An arts-and-health approach values the material world, relations with the material world and the satisfactions, through aesthetic appreciation and the recognition of others, that derive from using and creating good-quality artefacts. In this, the children were enabled to explore and develop their artistic abilities through the support of the arts practitioners who were themselves successful professional artists. Building communitas made the sessions a safe space for sharing and receiving compliments from their peers which translated into a growing confidence in their ability to produce work worth the attention of others:

‘Clare (who is shy and convinced she's rubbish at everything) … said “can you ask everyone to look at mine, it's well good!’. (Alice, 10/2/10).

However, although creating a quality product was part of the session aims, the children encountered the creative arts in a very different way to the everyday classroom; this was particularly evident in the writing-based intervention at Pennington. The arts practitioners effectively balanced the tensions between different forms of arts engagement. Too much playing and silliness would undermine the potential of art-making to explore emotions or enhance self-esteem; too much emphasis on techniques and product would make the sessions indistinguishable from classroom learning.

### 3.4 Transferability, integration and sustainability

Managing liminality involves balancing the integrity and permeability of boundaries. Contemporary settings of liminality, with their flexible temporalities are vulnerable to intrusions from the wider context in ways that Turner's settings of total and protracted withdrawal were not. Some intrusions from school or home, such as children arriving in heightened emotional states, were the proper business of the sessions and, whilst at times disruptive, part of a necessary permeability to the boundaries of the liminal time-space. However, other activities and priorities of the school also intruded and disrupted:

‘Mr. Green has asked how often the group would meet because Joshua is missing important science. J wants to stay in group but I think that discussion gave him some bounce.’ (Michael, 09/02/09).

Participants were sometimes absent because of timetable clashes with other activities, such as swimming, or even another extra-curricular intervention and the arts practitioners, as outsiders, were largely powerless in the face of this. At the same time, transformations within a liminal time-space are only of value if transferable back into the everyday. Thus, although session boundaries needed to be distinct and resistant to intrusion from the everyday life of the school, they also had to be sufficiently permeable for changes in children's self-narratives to be transferred, integrated and sustained into and within that everyday life (Kesby, 2007). In Turnerian liminality, re-integration back involved defined rituals. At Brightfields, Alice and teachers invited the children to an ‘Inside Me’ party as a fun and ritualised closure. At Pennington, some groups had parties or a reunion and most reflected on their experiences during the last session. But for some, time ran out, they had to leave the room and only managed a final debrief and farewell in the corridor. Even so, many asserted a strong sense of group identity:

‘We'll still be the Tarzan Poets, even if Michael's not here with us. And we'll go on to Secondary School together’ ‘Let's write a poem about each other’. (Documented by Michael, 10/02/09).

Any internal gains in children's emotional and social wellbeing would only be transferable and sustained beyond the liminal sessions if accorded recognition by others in their everyday worlds (Fisher, 2008). Both schools enabled formal presentations of work: Brightfields screened one group's animation film; a Pennington group sent their collection of poems to the Queen. The dedicated display board at Pennington was particularly significant; it was unlike any other in school filled only with children's work who themselves constantly referred to it. One teacher called it ‘a revelation’. At Pennington, the enjoyment of playing with words also benefited the children's engagement with the classroom literacy agenda:

‘I would like to make more stories. Before I needed help with literacy. Now I don't so much. I didn't use to ‘get’ playscripts, but I do more now.’ ‘Before, I didn't understand the work in class. Now I think I'm one of the best.’ (documented by Michael, 02/03/09).

Children took the initiative in presenting their work in assemblies and to their parents. Staff feedback at both schools reflected different temporalities of transformation, giving testimony to a mixture of dramatic transformations in classroom behaviours:

‘At the beginning of term, she wouldn't respond … but now, she seems to have just blossomed.’ (Brightfields staff, 06/09).

as well as slower, cumulative gains,

‘I'm glad she's been able to be part of the project over three terms, I really think she needed that amount of time’. (Alice, 24/6/09).

Liminality was created through strategies of separation and entry rituals, building communitas and the differentiated practices of power and artistry but the boundaries needed continual reinforcement and protection. Yet again, the need for the arts practitioner to facilitate a balance emerged, in this case between policing the boundaries and allowing permeability. Intrusions from the wider school constantly threatened the integrity of this time-space. However, if the boundaries had been too tightly protected, the role of the liminal time-space would have become merely one of sanctuary with limited potential for gains to be transferred and integrated back into the everyday emotional and social worlds of the participants.

## 4 Discussion

The practices of targeted arts-based interventions in schools manifest all the characteristic elements of liminality: displacement from, and dissolution of, the everyday classroom routines; building communitas within the targeted group; engagement in a guided set of arts-based activities in the form of tasks, rituals and games; re-integration and the sustained transferability of wellbeing gains back into the classroom. The arts practitioner is central as the mentor and guide who activates these elements to realise the transformative potential of a liminal time-space. In keeping with other contemporary accounts of liminality, a more flexible temporality did not undermine the development and delivery of the other defining elements.

As a practice of liminality, targeted arts-based interventions can generate the relational and transitional spaces, characterised by security, social integration, therapeutic experiences and capabilities (Fleuret and Atkinson, 2007), within which resources may be built and mobilised to effect the gains in a situated personal wellbeing (Kesby, 2007; Winnicott, 1971). The separation and dissolution of routines disrupt stable and habituated narratives whilst the processes of creative engagement generate trust and supportive relationships through communitas, the spaces of security and social integration in which to risk sharing and exploring. But although facilitating these spaces may seem a pre-requisite to those of capabilities and therapeutic processes, provoking openness through the arts to alternative possibilities to expand capabilities and to connect with emotions therapeutically are equally pre-requisites for trust and communitas. Thus, the elements involved in liminality facilitate the spaces of wellbeing as a complex process of mutually constituting interactions.

The classic description of liminality by Turner was informed by research in highly structured pre-industrial societies on equally highly structured practices of ritual and liminality. Turner himself limited the direct application of his theory to contemporary industrial societies, proposing the term ‘liminoid’ for settings where entry and participation are optional (Turner, 1974). The notion of an elective, fun, liminoid space of experience found intellectual purchase in the early ‘nineties, particularly within studies of tourism (Crang, 2005), whilst others reject both terms in favour of concepts to better convey heterogeneity and complexity, such as heterotopia (St John, 2001). Over the last ten years, framing contemporary settings through liminality has enjoyed a renaissance enabled by blurring the distinction between liminal and liminoid and allowing flexible temporalities. The modern English primary school with its national curriculum is comparable to the highly structured settings informing Turner's original thesis. Dislocation from this setting and the dissolution of its structured rules and practices may constitute a ‘classic’ Turnerian liminality and indicate continued relevance for the conceptual distinction of liminal and liminoid.

Whereas for Turner, the liminal phase enacted pre-defined practices for a pre-scripted transition, the practices enacted through the creative arts provoke the openness to ‘trying out’ new perspectives and identities described through liminality in other contemporary contexts (Madge and O'Connor, 2005). But whilst the practices and pathways of transformation are flexible, liminoid, these nonetheless reflect a pre-scripted outcome within a society in which self-realisation, self-actualisation and self-responsibilisation define the desirable modern citizen (Miller and Rose, 2008). Moreover, sustaining gains in wellbeing necessitates the recognition of transformation within the dominant developmental framing of the school system in which the pathways of a recognised successful becoming are indeed pre-scripted. The arts practitioner may thus be faced with tensions between managing both liminal and liminoid processes to enable children to negotiate both pre-scripted and creative explorations of their beings and becomings.

## 5 Conclusion

The field of arts-and-health draws on social concepts to understand its practice, distinct from the influence of psychological and psychoanalytical theories in art therapy. But both fields have neglected the spatialities of the processes in their practice through which participation in the creative arts may benefit personal wellbeing. The paper foregrounds the spatialities of arts-based practice and argues that, in the school setting, arts-based interventions are usefully understood through a framing of liminality. The practice of the artist in managing the liminal time-space is critical as they must negotiate balances between several inherent tensions. Framing arts-based practices as a spatial practice connects the field theoretically into both the social and spatial sciences and the psychoanalytic tradition of Winnicott's transition spaces (1971), offering a potential pathway towards theorisation that can bridge the diversity of practice in engaging the arts to enhance health and wellbeing.
